# Climate change-based models predict range shifts in the distribution of the only Asian plethodontid salamander: *Karsenia koreana*

**DOI:** 10.1038/s41598-019-48310-1

**Published:** 2019-08-14

**Authors:** Amaël Borzée, Desiree Andersen, Jordy Groffen, Hyun-Tae Kim, Yoonhyuk Bae, Yikweon Jang

**Affiliations:** 1 Department of Life Science and Division of EcoScience, Ewha Womans University, Seoul, 03760 Republic of Korea; 2 Interdisciplinary Program of EcoCreative, Ewha Womans University, Seoul, 03760 Republic of Korea; 3 Department of Fish and Wildlife Conservation, Virginia Tech, Blacksburg, Virginia 24061 USA; 4 Seosan Joongang High School, Seosan, South Chungcheon Province Republic of Korea; 5 Department of Life Science, College of Natural Science, Hallym University, Chuncheon, Republic of Korea

**Keywords:** Climate-change ecology, Projection and prediction

## Abstract

Populations see their range fluctuate in relation to environmental variations, including climate change, and their survival is linked to the maintenance of large enough populations and broad enough distributions during these variations. Most amphibian populations are threatened by numerous ecological and anthropogenic variables acting in synergy with climate change. Accumulating basic ecological data such as range enables the development of population and range dynamics, themselves resulting on adequate conservation plans. *Karsenia koreana* is the only known Asian plethodontic salamander, occurring in a very restricted area only. Based on presence data, we created an ecological model using six bioclimatic factors with low multicollinearity to define the adequate habitat of the species, and we modelled the predicted suitability of the Korean landscape following four Representative Concentration Pathways (RCPs) predicting climate change scenarios based on CO_2_ concentrations in 2050 and 2070. The maximum entropy model for the current distribution produced a landscape suitability considerably wider than the current known distribution. The projected ranges for each RCP indicated marked increases, decreases and shifts in areas with suitable landscapes due to climate change. The lowest RCP prediction resulted in an increase in suitable area, although potentially without connectivity with current populations, while the highest RCP predictions resulted in a decrease. Our results highlight the potential negative impact of climate change, thus requiring updates in conservation plans for *K. koreana*. The methods used here can be replicated with any land-dwelling species, and our results reflect expected range shifts for most amphibians of the northern hemisphere.

## Introduction

Range variations due to climate change have been described in several species and tend to result in the shift of climate envelopes towards the poles and higher elevations^1^. For instance, a three degree Celsius increase matches with a 350 km shift in latitude, or a 500 m shift in altitude^2^. Patterns of range shifts resulting from climate change have been well documented in migratory species despite large yearly variations^1,3,4^, and a wide range of taxa displays such a range displacement (reviewed by Hughes, 2000^5^). Several studies demonstrated a range shift for 22 species of non-migratory European butterflies^6^, Edith’s checkerspot butterfly^7^, mosquito species in equatorial areas^8^, birds, anoline lizards^9^, 59 British bird species^10^, 14 North American bird species^11^, 19 North American mammals^12^, the bacterium *Pseudomonas syringae* in China^13^ and benthic species in the Mediterranean sea^14^. This is also the case for amphibians, with species such as red-eyed stream frogs (*Hyla uranochroa*) predicted to shift distribution as a result of climate change^9^.

Species distribution models (SDMs) are spatial representations of species presence probability or abundance. They are created using responses to environmental predictor variables, spatial relationships such as convex hulls or interpolation, or a combination of the two. SDMs are easily projected across landscapes, and can therefore be helpful in circumstances where acquiring occurrence data on the distribution of species is not achievable, or when the ecological variables related to the distribution of the species have changed (reviewed by Guisan and Thuiller 2005^15^). The change may result from long-term shifts in environmental variables, as illustrated by the range shift between the paleo and current distributions of the Japanese Treefrog, *Dryophytes japonicus*, in North East Asia^16^. SDMs can also be used to predict variations in the distribution of species due to future anomalies resulting from climate change^9,13,14,17–19^. Ecological models thus allow determining the range of species in conditions where it would not be otherwise doable or when the range boundaries are not known^20–22^. For instance, eight new localities of *Vipera ursinii graeca* were found through landscape and climate modelling, doubling the known range of the species^23^. Spatial modelling for some clades without clear geographic boundaries, such as fish or birds^24^ is inherently more complex. However, species like amphibians are perfect model species as they do not disperse over long distances^25^, nor do they migrate over numerous climatic zones, and thus have generally continuous distribution patterns^26^.

Amphibians are comparatively more endangered and less studied than their counterparts, be them flying or apex predator^27^, and the range of most species is not accurately defined, with many species classified as Data Deficient under the IUCN criteria^28^. This is especially true for geographic areas where ecological research is less widespread. For instance, the Suweon Treefrog (*Dryophytes suweonensis*) in the Republic of Korea saw its known range doubled between 2012^29^ and 2017^30,31^.

Here we focus on the only plethodontid salamander in Asia, the Korean Crevice Salamander, *Karsenia koreana*. It is considered one of the most elusive Korean amphibian species, and although it was first collected in 1971^32^, it was only described in 2005^33^. Accordingly, very little is known about its morphology^34–37^ and genetics^38–41^. In additions, *K. koreana* is not stringently bound to the aquatic environment as a result of the reacquisition of its larval stage^42^ and unusual breeding behaviour^43,44^. Little or no information is available about the potential range shift for *K. koreana* due to climate change^45^, but the latter is expected to have a negative impact on habitat suitability for plethodontids in general^46–48^. Climate change has likely caused body size reductions in other plethodontid salamander species^49^, which affects fecundity (reviewed by Wells, 2007^50^) and rates of water loss^51,52^. In turn this may impact surface activity time^53^, and consequently affect foraging, dispersal, reproduction, and might change future species’ distributions^54^. As the climate on the Korean peninsula is getting warmer^55,56^ and dryer^57^, *K. koreana* is also going to be affected by climatic variations and related ecological changes. The aims of this study were (1) to define the potential current distribution of *K. koreana* through the development of a habitat suitability model and a set of bioclimatic and vegetation variables; (2) to investigate the potential impact of climate change on the distribution of *K. koreana* in the Korean peninsula using four Representative Concentration Pathways (RCPs), which are climate change scenarios based on greenhouse gas concentration trajectories.

## Material and Methods

### Species and habitat

Very little is known about *Karsenia koreana*. Its breeding behaviour was observed for the first time in 2016, but it still lacks any formal description in the wild^44^. The species is not as stringently bound to the aquatic environment as other amphibians due to the absence of a larval stage^42^, and to unusual breeding behaviour^43,44^. Like most plethodontids, *K. koreana* breeds and lays eggs on land and where they develop directly into adults without a larval stage^34^. Similarly, very little is known about the morphology^34,35,37^ or the genetics of the species^38–41^.

Until recently, *K. koreana* had only been found at a few locations under rocks on moist hills of montane woodlands with limestone soils^33^, while other plethodontid salamanders are described in a range of different habitats *e.g*., aquatic, fossorial, arboreal, stream, terrestrial or cave^58^. The behaviour of *K. koreana* in relation to habitat use is not known, although Buckley, *et al*.^34^ suggested the use of shelters in tight spaces between rocks, based on the species’ skull morphology.

### Field survey

**Figure 1:**
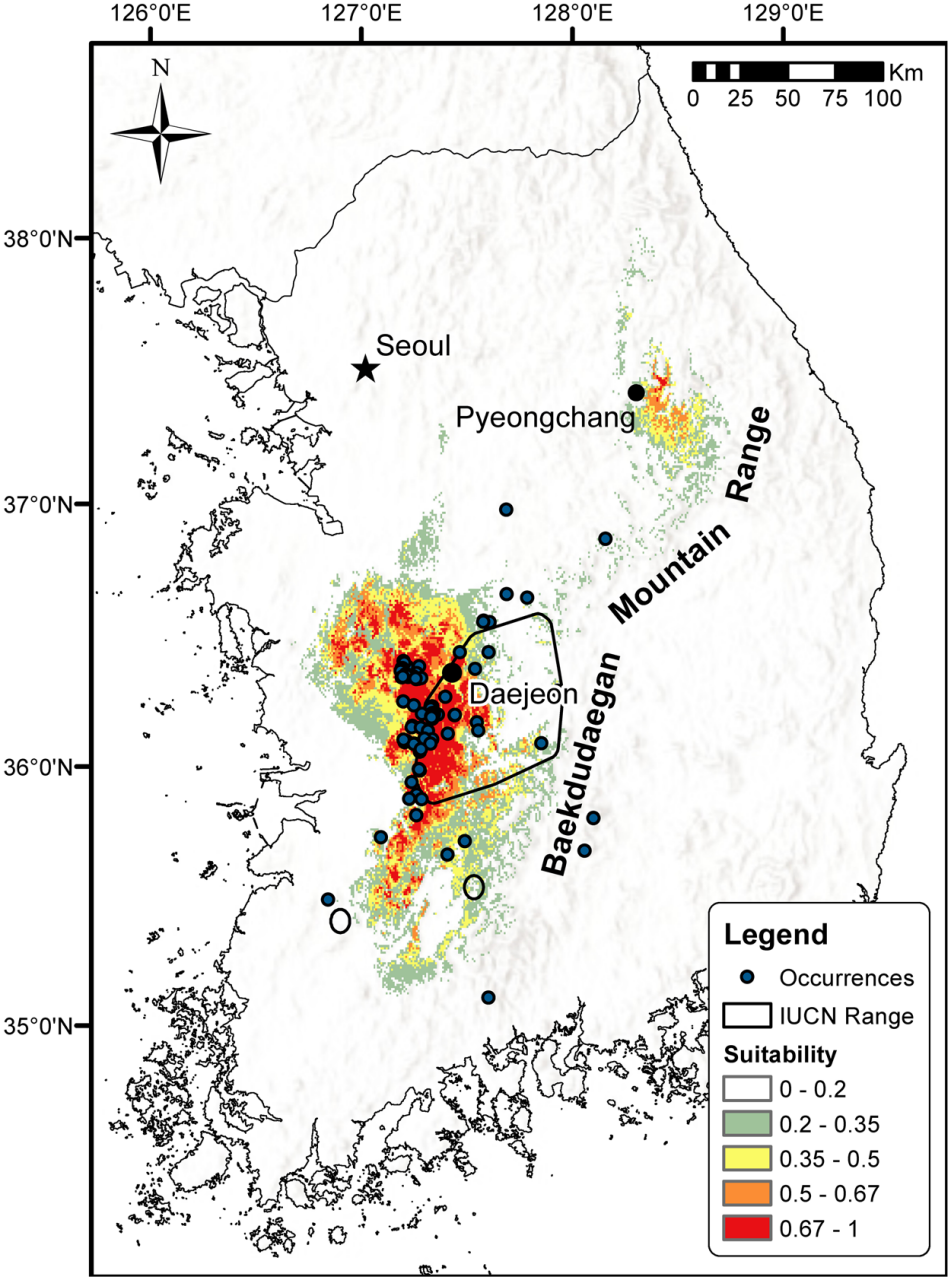
Map of the Korean peninsula showing habitat suitability for *Karsenia koreana* based on 19 bioclimatic variables. Red areas indicate higher suitability. Observation points and IUCN range are also shown. Map generated in ArcMap 10.6.

A total of 143 opportunistic field surveys were conducted between the first week of April 2007 and the last week of August 2017. Initial surveys were focused around Daejeon (Republic of Korea; Fig. 1), the area where the species was first found (2007–2008), and later expanded to similar types of habitat (2009–2016). Each observation was recorded with date, time and GPS coordinates (latitude and longitude).

### Spatial model, current suitability and field validation

We used Maxent version 3.4.0^59^, the maximum entropy approach (http://biodiversityinformatics.amnh.org/open_source/maxent/, accessed April 2018) to assess the potential distribution of *K. koreana* using species’ occurrences and environmental variables to develop habitat suitability maps. The reliability of the prediction depends on the variables selected and their resolutions. The 19 bioclimatic variables traditionally used for SDM^60–63^ were tested for multicollinearity using a Pearson’s correlation test, and the variables retained in model testing (Table 1) included Bio2 (mean diurnal range), Bio3 (isothermality), Bio4 (temperature seasonality), Bio6 (minimum temperature of coldest month), Bio14 (precipitation of driest month), and Bio16 (precipitation of wettest quarter). All variables were represented by rasters with resolutions of 30 arc seconds (~1 km) and had Pearson’s correlation coefficients less than 0.8. These variables were selected to represent climatic limitations, such as minimum and maximum temperature and precipitation, as such variables have been shown to have physiological implications for individual survival^64,65^.

**Table 1:** Bioclimatic variables (Hijmans *et al*. 2005) used as Maxent environmental factors and in RCP projections to model the current and future habitat suitability for *Karsenia koreana*.

Bioclimatic Variable	Description
BIO2	Mean Diurnal Range (Mean of monthly (max temp-min temp)
BIO3	Isothermality ((BIO2/BIO7)*100)
BIO4	Temperature Seasonality (standard deviation*100)
BIO6	Min Temperature of Coldest Month
BIO14	Precipitation of Driest Month
BIO16	Precipitation of Wettest Quarter

To determine the current suitability range for *K. koreana*, Maxent was run with 82 occurrences, reduced from 139 by removing duplicate presence records within 1 km^2^ to reduced spatial autocorrelation caused by sample bias^21,66^. Ten bootstrap runs with a 30 percent random test percentage were output as cloglog, which is considered appropriate for estimating presence probability^59^, with a threshold at 10-percentile training presence. The averages of all runs were used as final models, and jackknife analysis was used to determine the factors contributing the greatest amount to habitat suitability. The final model was evaluated by area under the curve (AUC), true skill statistic (TSS^67^) and percent of occurrences within the 10-percentile training presence area. Additionally, we verified the accuracy of our prediction by comparing predicted suitable areas with the species’ reported distribution^68,69^.

### Projected suitability

We predicted the potential future distribution of *K. koreana* using four climate change scenarios or Representative Concentration Pathways (RCPs) based on atmospheric CO_2_ trajectories. These four RCPs correspond to increases of 2.6, 4.5, 6.0, and 8.5 watts/m^2^ by 2100, with wattage increasing with projected CO_2_ concentrations (Table 2)^70–73^. SDMs were developed for two time-steps: 2050 (average for 2041–2060) and 2070 (average for 2061–2080) for all four RCPs. Among the global circulation models (GCMs) we used for all projections the Community Climate System Model 4.0 (CCSM4) from the Coupled Model Intercomparison Project Phase 5 (CMIP5) developed by the Intergovernmental Panel on Climate Change (IPCC) at 30 arc seconds, or approximately 1 km^2^ resolution, a choice confirmed by the jackknife analysis^74–76^.

**Table 2:** Climate scenarios RCP 2.6, 4.5, 6.0, and 8.5 used to predict the range of *Karsenia koreana* in 2050 and 2070.

Scenario	Radiative forcing increase (watts/m^2^ increase in 2100)	Year of peak greenhouse gas emissions	Decline during 21^st^ century?
2.6	2.6	2010–2020	Yes
4.5	4.5	2040	Yes
6.0	6.0	2080	Yes
8.5	8.5	n/a	No

## Results

### Current suitable range

The results of our SDMs (Fig. 1) showed a marked increase of potential suitable range for *Karsenia koreana* in respect to that described by previous studies^45^. The IUCN range did not include the areas with high suitability in the northwestern and southern regions of the country. The potential distribution resulting from our models covered a large part of western Korea, situated between ca. 100 and 700 meters above sea level west of the Baekdudaegan Mountain Range (Fig. 1). There is also a disjunct area of high suitability located to the northeast in the Pyeongchang area, which matches with reports of the species in that region^68^.

The Maxent modelling of current climate conditions (Fig. 1) produced a range of suitable habitat (cloglog output above the 10-percentile training presence threshold of 0.1964) was 10,261 km^2^. This model had an averaged AUC of 0.984 ± 0.007 and TSS of 0.869 ± 0.017 and could therefore be accepted as a model for the species’ habitat suitability. Suitable area thresholded at the 10-percentile training presence covered 93.6% of total presence points including 14 of the 15 counties, cities, and districts reported in The Encyclopedia of Korean Amphibians^68^ and at most locations reported in the Arrow guide of amphibians and reptiles^69^.

### Climate scenario projections and jackknife analysis

**Figure 2:**
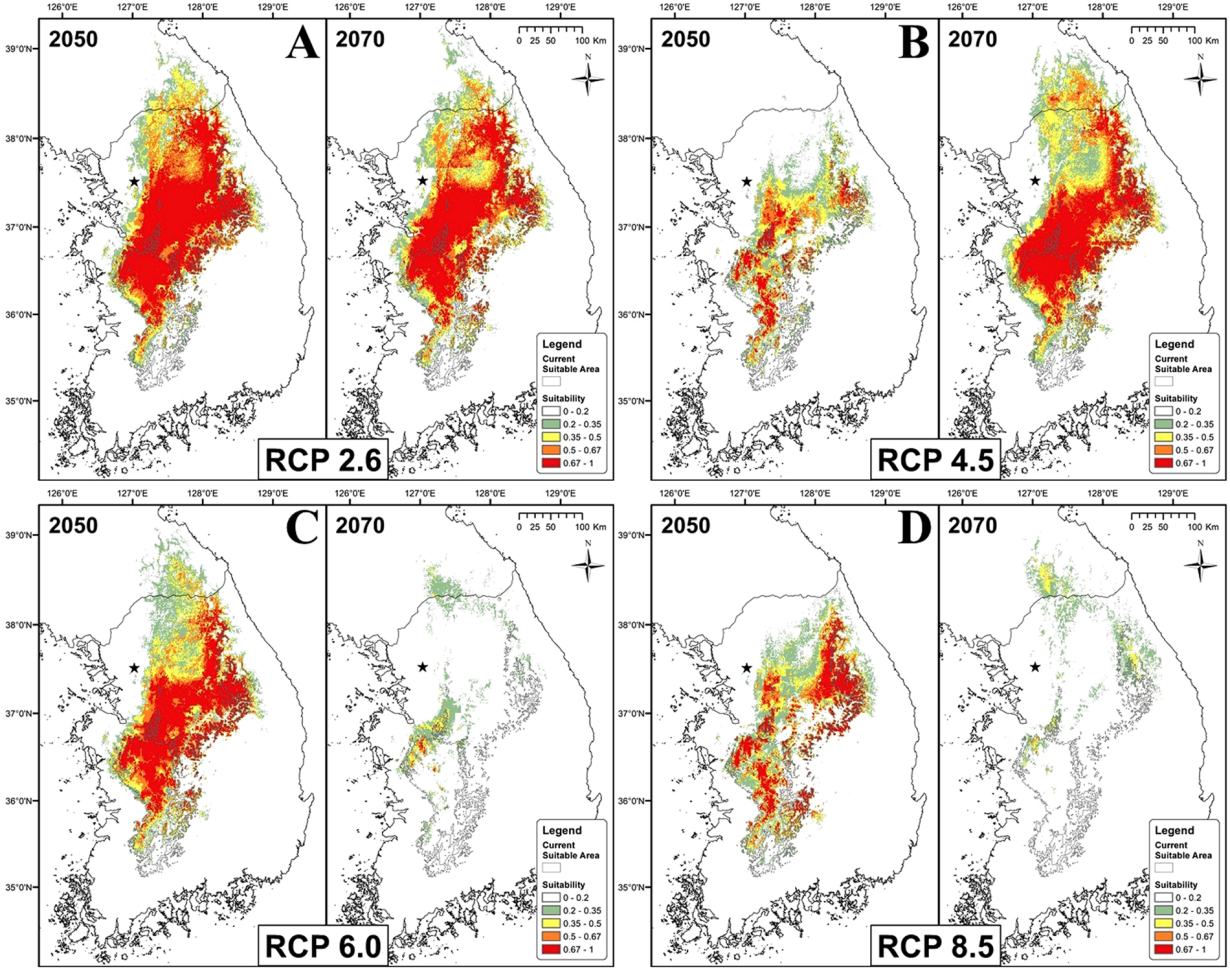
Projected suitable range for *Karsenia koreana* in RCP 2.6 (**A**), RCP 4.5 (**B**), RCP 6.0 (**C**), RCP 8.5 (**D**) for 2050 and 2070. Map generated in ArcMap 10.6.

**Figure 3:**
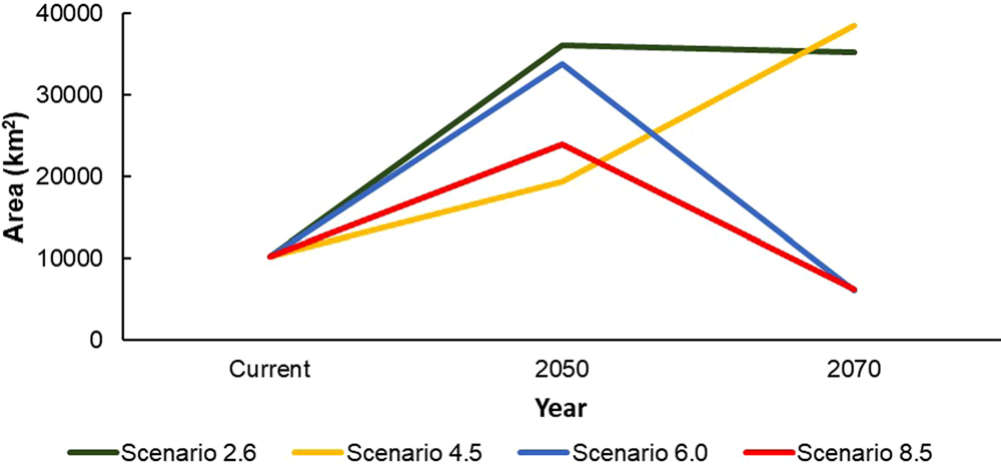
Comparison for the area of suitability (>0.1964) for *Karsenia koreana* based on four RCP scenarios. Suitable area in km^2^.

The climate projections showed a shift of the current suitability range for the species towards the northern and eastern regions, characterized by higher altitude (Fig. 2, Table 3). Following the RCP 2.6 and 4.5 predictions, the area of suitability will increase to the north and to higher elevations through 2070, while RCPs 6.0 and 8.5 show increases by 2050 but marked decreases by 2070 (Fig. 3). The two latter RCPs are clearly negative for the habitat suitability of *K. koreana*, highlighting a significant loss of suitable habitat by 2070.

**Table 3:** Suitable area in km^2^ and percentage of current occurrences within suitable area for *Karsenia koreana* in the Republic of Korea under four climate change scenarios in 2050 and 2070.

Scenario	2050		2070	
	Area (km^2^)	Percentage	Area (km^2^)	Percentage
RCP 2.6	36.033	96.4	35.172	96.4
RCP 4.5	19.417	95.7	38.506	96.4
RCP 6.0	33.744	97.1	6.156	25.9
RCP 8.5	23.947	95.0	6.219	18.0

**Figure 4:**
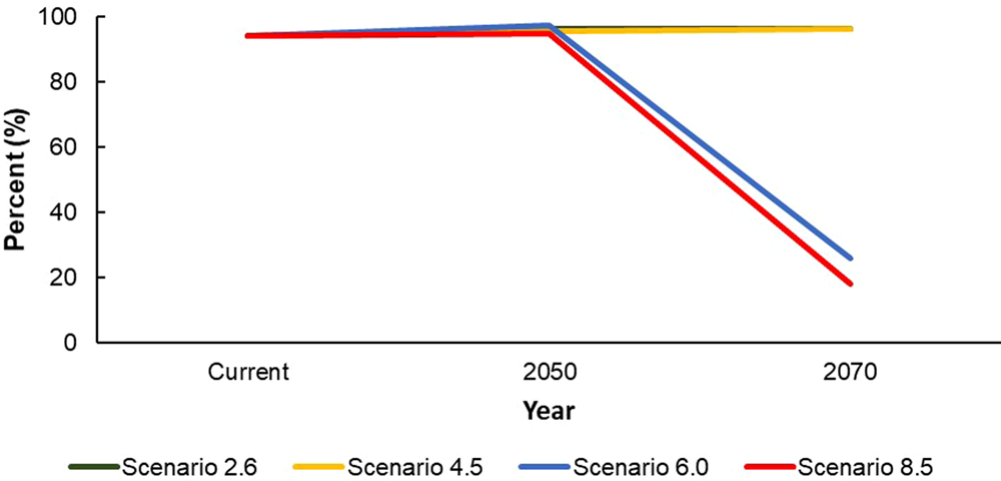
Percentage of current observation locations present in suitable areas (>0.1964) for *Karsenia koreana* under the four RCPs.

In all RCPs, suitable area is expected to increase by 2050. However, for RCPs 6.0 and 8.5, suitable area will decrease significantly by 2070. The most favorable scenarios for suitable habitat (RCPs 2.6 and 4.5) resulted in increases of 243% and 275%, respectively, in suitable habitat area by 2070, while the less favorable scenarios (RCPs 6.0 and 8.5) resulted in decreases of 40% and 39%, respectively, in suitable area by 2070 (Table 3, Fig. 3). Similarly, occurrence points located within suitable areas will be maintained through 2070 for RCPs 2.6 and 4.5 but will fall to 25.9% and 18.0% in RCPs 6.0 and 8.5, respectively (Table 3, Fig. 4). Here, higher percentages indicate a lower range shift for the species, while lower percentages indicate a higher range shift.

**Figure 5:**
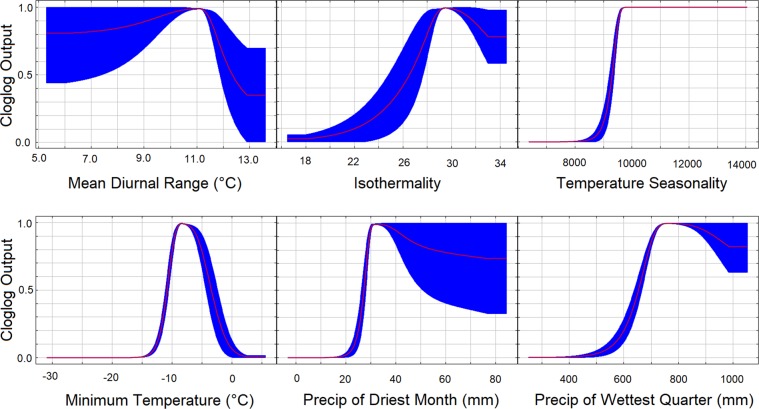
Response curves of *Karsenia koreana* to bioclimatic variables used in Maxent modelling.

In jackknife analysis (Fig. 5), the three highest-contributing bioclimatic variables in order were Bio14 (precipitation of driest month), Bio4 (temperature seasonality), and Bio3 (isothermality). Similarly, Bio14 had the highest contribution and second highest permutation importance, the response to which was an increase in presence probability with precipitation in mm during the driest month. The variables with the lowest contributions were Bio2 (mean diurnal range), Bio6 (minimum temperature of coldest month), and Bio16 (precipitation of wettest quarter). However, Bio6 had the highest average permutation importance.

## Discussion

Our results highlight a significantly larger habitat suitable for *Karsenia koreana* than is the current known distribution of the species^45,68^. The fact that the model matches with the known presence in counties^68^ and five other data points^77–81^. highlights the accuracy of our prediction. Discrepancies are not impossible as precise spatial modelling from a low number of samples can result in large variations^82^. The range of the species may be restricted by additional factors that could not be included in the model, such as competition. As an example, within plethodontid salamander, spatial distributions can be influenced by interference competition to complete competitive exclusion^83–85^. Second, salamanders may have been extirpated from some parts of the range, for instance during the Korean War when logging was intensive. However, Plethodontids are lungless ectotherms, and it appears that their activity and geographic distribution is predominantly controlled by climate^86–88^. Finally, salamanders may exist in areas where they have not been discovered yet, which introduces a bias in the ecological preferences of the species, and on the SDM as a result. Additional field surveys are required to clearly record the occurrence of the species, and further observations would improve the precision of habitat suitability models.

Despite this potentially larger new range, the threat status of *K. koreana* is still unresolved, as species with a limited range have often larger projected habitat loss and are more prone to extinction^48,89,90^, and a present point of concern in the Republic of Korea^91^. The results of Maxent modelling identify areas that are potentially important habitats for *K. koreana* and provide researchers with new potential survey locations. These results can inform future conservation efforts for the species in terms of protecting important habitat areas; the ranges calculated for habitat suitability fall within different threat levels of the IUCN red list of endangered species^92^, and *K. koreana* would thus join the list of endangered species from the Korean Peninsula^93^. Additionally, acquiring the exact area of occupancy of the species would be important for ecological monitoring as *K. koreana* is likely to be sensitive to the newly emerged Chytrid fungus *Batrachochytrium salamandrivorans*^94^. This fungus may soon be present in the Republic of Korea because of the pet trade, the same way it found its way to Europe^95^, and the same way the anuran Chytrid fungus entered Korea^96^.

Studies evaluating the effect of climate change on amphibian populations are based on both breeding phenology^97,98^ and range shifts^9^. Furthermore, the impact of agriculture on amphibian assemblages is well-studied and has been found to have two main types of impacts: landscape fragmentation (reviewed by Cushman 2006^99^) and chemical use (reviewed by Mann *et al*.^100^). The predicted range of suitability resulting from Maxent modelling is an increase of 80% from the current IUCN range of 5,687 km^2^ (or 1.8 times the IUCN range). This area contains 93.8% of the observed points, so it can be considered an improvement of the IUCN range, which only contains 51.4% of the observed points. Because some of the predicted areas are fragmented or too small, or because the species may not manage to disperse there, it is likely that the actual range will be smaller than the predicted 10,261 km^2^. Additionally, there are areas with medium to high suitability where the species has yet to be recorded.

The jackknife analysis indicates that precipitation is likely a limiting environmental factor in the range of *K. koreana*, and that the species requires sufficient precipitation in the dry winter months to persist. Projections for all RCPs indicate that future habitats for *K. koreana* will be fragmented. In all cases, the suitable range for the species will shift, in which case the species may require translocation to persist. With continued development of natural areas by humans, translocation to more remote areas that already have protected status may be necessary. For instance, Baekdudaegan Mountains Reserve is currently outside the suitable range for *K. koreana*, but projections indicate the species may be able to inhabit areas in the reserve in the future. Since the goal of this modelling was to determine effects of climate change on *K. koreana*, this model leaves out other potentially important environmental factors, such as greenness and wetness. These factors will likely change along with the climate, and therefore, current maps of these factors could not be used in projections for this study.

## Data Availability

All data used for the analyses will uploaded to an online data repository upon acceptation of the manuscript.
